# Lipid nanoparticles for transdermal delivery of flurbiprofen: formulation, *in vitro, ex vivo *and *in vivo *studies

**DOI:** 10.1186/1476-511X-8-6

**Published:** 2009-02-26

**Authors:** Kesavan Bhaskar, Jayaraman Anbu, Velayutham Ravichandiran, Vobalaboina Venkateswarlu, Yamsani Madhusudan Rao

**Affiliations:** 1 Department of Pharmaceutics, School of Pharmaceutical Sciences, VELS University, Velan Nagar, Pallavaram, Chennai- 600 0117, Tamil Nadu, India; 2 Novel Drug Delivery Systems Laboratory, University College of Pharmaceutical Sciences, Kakatiya University, Warangal-506 009, Andhra Pradesh, India

## Abstract

The aim of the study is to prepare aqueous dispersions of lipid nanoparticles – flurbiprofen solid lipid nanoparticles (FLUSLN) and flurbiprofen nanostructured lipid carriers (FLUNLC) by hot homogenization followed by sonication technique and then incorporated into the freshly prepared hydrogels for transdermal delivery. They are characterized for particle size, for all the formulations, more than 50% of the particles were below 300 nm after 90 days of storage at RT. DSC analyses were performed to characterize the state of drug and lipid modification. Shape and surface morphology were determined by TEM which revealed fairly spherical shape of the formulations. Further they were evaluated for *in vitro *drug release characteristics, rheological behaviour, pharmacokinetic and pharmacodynamic studies. The pharmacokinetics of flurbiprofen in rats following application of SLN gel (A1) and NLC gel (B1) for 24 h were evaluated. The C_max _of the B1 formulation was 38.67 ± 2.77 μg/ml, which was significantly higher than the A1 formulation (C_max _= 21.79 ± 2.96 μg/ml). The C_max _and AUC of the B1 formulation were 1.8 and 2.5 times higher than the A1 gel formulation respectively. The bioavailability of flurbiprofen with reference to oral administration was found to increase by 4.4 times when gel formulations were applied. Anti-inflammatory effect in the Carrageenan-induced paw edema in rat was significantly higher for B1 and A1 formulation than the orally administered flurbiprofen. Both the SLN and NLC dispersions and gels enriched with SLN and NLC possessed a sustained drug release over period of 24 h but the sustained effect was more pronounced with the SLN and NLC gel

## Background

Drug delivery from colloidal systems such as solid lipid nanoparticles (SLN) and nanostructured lipid carriers (NLC) dispersed in a hydrogel appears to be unique when compared to the delivery from traditional topical and dermatological formulations. During the last decade, considerable attention has been paid to the development of new controlled delivery systems, in order to supply a long-term drug release and, therefore, increase patient's therapeutic compliance and acceptance. SLN and NLC are interesting systems for the present purpose due to their solid matrix which might avoid the burst release obtained in conventional topical formulations [1,2]. These lipid nanoparticles differ from each other because in NLC a controlled nanostructuring of the lipid matrix is performed due to the mixture of solid and liquid lipids, in order to increase drug-loading and prevent its expulsion. In addition, the nanostructured lipid matrix gives more flexibility in modulation of drug release.

Solid lipid nanoparticles (SLN) and nanostructured lipid carriers (NLC) are two main types of lipid nanoparticles. SLN is a colloidal carrier system for controlled drug delivery, followed by the development of emulsion, liposomes, microparticles and nanoparticles based on synthetic or natural polymers [3]. They combine the advantages of emulsions, liposomes and polymeric nanoparticles. The solid matrix can protect incorporated active ingredients against chemical degradation and provide the highest flexibilities in the modulation of the drug release profiles. Moreover, the SLNs are composed of physiologically well tolerated excipients. Advantages of SLN and NLC include a potentially wide application spectrum (dermal, oral, intravenous), the use of biodegradable physiological lipids or lipidic stabilizers which are generally recognized as safe (GRAS) or have a regulatory accepted status. NLC composed of solid lipid matrix with certain content of liquid lipid are a new generation of lipid nanoparticles. The incorporation of liquid lipids into solid lipid matrix leads to great imperfections in the crystal lattice of nanoparticles, thus leading to improved drug loading capacity and reduced drug expulsion during storage [4,5].

As transdermal delivery system, SLN and NLC possess many obvious advantages. It is assumed that SLN and NLC, when administered directly onto skin in a small but sufficient quantity, would cause less side effects, if any, than the currently available formulations. SLN and NLC seem to be well suited for use on damaged or inflamed skin because they are based on non-irritative and non-toxic lipids. With respect to their use as carriers for topical applications, the occlusive effect due to film formation on the skin surface that reduces transepidermal water loss (TEWL). Increasing the water content in the skin can reduce the symptoms of atopic eczema and improve the appearance of healthy human skin. Occlusion can also enhance the penetration of drugs through the stratum corneum by increased hydration. Apart from a nonspecific occlusion effect on penetration, penetration might also be affected by the SLN and NLC carrier itself, the high specific surface area of nanometer sized SLN and NLC facilitates contact of encapsulated drugs with the stratum corneum [4].

The main purpose is to investigate the latest developments of innovative solid lipid carriers, particularly solid lipid nanoparticles (SLN) and nanostructured lipid carriers (NLC), for transdermal delivery of flurbiprofen.

Flurbiprofen is a chiral non-steroidal anti-inflammatory drug (NSAID) of the 2-arylpropionic acid class. Flurbiprofen, one of the most potent inhibitors of platelet aggregation currently available, is used to treat gout, osteoarthritis, rheumatoid arthritis, and sunburn. Flurbiprofen has also been found to cause a dose-dependent inhibition of collagen-induced platelet aggregation in platelet-rich plasma from human, rats and rabbits *in vitro*. Upon oral administration, the most frequently reported side effects of flurbiprofen are abdominal discomfort along with other gastrointestinal effects. Also, it has a short elimination half-life of 3.9 h and requires frequent dosing. Therefore long-term percutaneous absorption of flurbiprofen at a controlled rate is needed. Transdermal delivery would provide a means to avoid the gastrointestinal damage associated with oral route. However, intercellular lipid barrier in the stratum corneum has formidable barrier properties and most of the drugs cannot penetrate the skin readily.

The aims of the present study were to 1) develop SLN and NLC enriched hydrogels for transdermal delivery, 2) perform *in vitro *and *in vivo *permeation studies through rat skin, and 3) evaluate the efficacy of SLN/NLC gels against inflammation induced rats. The purpose was to provide the delivery of the drug at a controlled rate across intact skin to improve bioavailability and inflammation control for longer period from SLN/NLC gels.

This paper was also designed to assess the possibility to develop SLN and NLC based semi-solid formulations using a well established hydrogel as transdermal vehicle. We have also tried to improve the bioavailability of the transdermally applied flurbiprofen. The objectives of this study were to develop a patient non infringing drug delivery system that could reduce the side effects due to optimization of the blood concentration time profile and to extend duration of activity which allows greater patient compliance owing to elimination of multiple dosing schedules.

## Methods

### Materials

The following materials were used from the indicated sources without further purification procedures. Flurbiprofen and ibuprofen were kindly gifted by Sun Pharmaceutical Industries LTD (Mumbai, India). Trimyristin (TM) (Dynasan 114) was generously supplied by Sasol (Witten, Germany); Captex 355 EP/NF (triglycerides of caprylic and capric acid) was donated by Abitec Corporation (Janesville, Winsconsin, USA), Soy phosphatidylcholine 99% (Epikuron 200) was donated by Degussa Texturant Systems (Deutschland, Hamburg, Germany). Tween 80 and dialysis membrane-70 were purchased from Hi-Media (Mumbai, India). For hydrogel preparation, Carbopol 934 (polyacrylate) was purchased from BF Goodrich (Cleveland, Ohio, USA), Xanthan gum was purchased from Sigma-Aldrich (Mumbai, India), hydroxy propyl cellulose (HPC) was purchased from Hi-Media (Mumbai, India). Chitosan (degree of deacetylation, 80.8%) was gifted by Central Institute of Fisheries Technology and India Sea Foods (Kochi, India). Centrisart filters (molecular weight cutoff 20,000) were purchased from Sartorius (Goettingen, Germany). The other chemicals were of analytical reagent grade.

### Preparation of aqueous SLN and NLC dispersions of Flurbiprofen

FLU (1% w/v), Dynasan 114 (5% w/v), and phosphatidylcholine 99% (2% w/v) were dissolved in 10 ml mixture of chloroform and methanol (1:1). Organic solvents were completely removed using a rotoevaporator (Laborota 4000, Heidolph, Germany). Drug-embedded lipid layer was melted by heating at 58°C above melting point of the lipid. An aqueous phase was prepared by dissolving polysorbate 80 (1% w/v) in double distilled water (sufficient to produce 10 ml of preparation) and heated to same temperature of oil phase. Hot aqueous phase was added to the oil phase, and homogenization was carried out (at 12,000 rpm and temperature 70°C) using a Diax 900 homogenizer (Heidolph, Germany) for 3 min. Coarse hot oil in water emulsion so obtained was ultrasonicated (12T-probe) using a Sonoplus ultrahomogenizer (Bandelin, Germany) for 15 min. Flurbiprofen solid lipid nanoparticles (FLUSLN) were obtained by allowing hot nanoemulsion to cool to room temperature.

Flurbiprofen nano-structured lipid carriers (FLUNLC) were prepared in exactly the same manner as the SLN dispersions, only partially replacing 30% of the solid lipid matrix by Captex 355 EP/NF (caprylic/capric triglycerides).

### Gels enriched with lipid nanoparticles

Gels were prepared using four polymers namely carbopol 934 (1%), xanthan gum (1%), hydroxyl propyl cellulose (2%) and chitosan (1%). For the preparation of hydrogel, the gel forming polymer was dispersed in double distilled water containing glycerol (10%). Aqueous FLUSLN and FLUNLC dispersions and hydrogels were mixed in a high speed stirrer (Remi, Mumbai, India) at approximately 1000 rpm for 5 min to yield gels containing a final concentration of 5% lipid nanoparticles. The hydrogels composed of carbopol 934 and chitosan were adjusted to pH 6.5 and 4.0 respectively. The SLN and NLC dispersions were used as reference. The SLN and NLC loaded FLU hydrogels were stored at room temperature for 90 days.

### Physicochemical Properties of SLN and NLC Hydrogels

The SLN and NLC enriched hydrogels were characterized for their physicochemical properties such as colour, odour and pH.

### Measurement of Particle size of SLN and NLC enriched hydrogels

The mean size and polydispersity index of the size distribution of FLUSLN and FLUNLC was determined by photon correlation spectroscopy using Zetasizer 3000 HSA (Malvern Instruments, Malvern, UK). The SLN dispersions were diluted 1:1000 with the aqueous phase of the formulation to get a suitable kilo counts per second (kcps). Analysis was performed at 25°C with an angle of detection of 90°. Each value reported is the average of three measurements. The polydispersity index measures the size distribution of the nanoparticle population.

### Measurement of zeta potential of SLN and NLC enriched hydrogels

Zeta potential of different formulations of flurbiprofen SLN and NLC were measured by Photon Correlation Spectroscopy (PCS) using Zetasizer 3000 HSA (Malvern Instruments, Malvern, UK). The SLN dispersions were diluted as mentioned in size determination.

### Assay and Entrapment Efficiency

The prepared FLUSLN/FLUNLC dispersions (0.2 ml) were diluted to 10 ml with chloroform/methanol (1:1). Final dilution was made with mobile phase, and FLU content was determined by HPLC.

The entrapment efficiency of the system was determined by measuring the concentration of free drug in the dispersion medium/aqueous phase of undiluted FLU/SLN dispersion. Ultracentrifugation was carried out using Centrisart, which consists of filter membrane (molecular weight cut-off, 20,000 Da) at the base of the sample recovery chamber. About 1 ml of undiluted sample of FLUSLN/FLUNLC dispersion was placed in the outer chamber, and the sample recovery chamber was placed on top of the sample. The unit was centrifuged at 10,000 rpm for 30 min. The SLN/NLC along with encapsulated drug remained in the outer chamber, and aqueous phase moved into the sample recovery chamber through filter membrane. The amount of the FLU in the aqueous phase was estimated by HPLC.

### HPLC analysis of Flurbiprofen

The mobile phase consisted of a 40% aqueous phase adjusted to pH 2.6 with acetic acid; and 60% acetonitrile. Mobile phase was degassed with the help of bath sonicator. The chromatographic system consisted of a Shimadzu LC-10AT solvent delivery pump equipped with a 20 μl loop and rheodyne sample injector. Phenomenex (25 cm × 4.6 mm ID) analytical column was used. Detector used was SPD-10A VP dual wavelength UV-Visible detector (Shimadzu) and the eluate was monitored at 245 nm. The sensitivity was set at 0.001 AUFS. Flow rate was kept at 1 ml/min. The data was recorded using Winchrome Software.

### DSC Analysis

DSC analysis of flurbiprofen (FLU), trimyristin (TM), physical mixture (PM), and lyophilized FLUSLN and FLUNLC were performed using Perkin-Elmer DSC-7 model. The instrument was calibrated with indium. All the samples (≈5 mg) were heated in aluminum pans using dry nitrogen as the effluent gas. The analysis was performed with a heating range of 20–200°C and at a rate of 20°C/min.

### High Resolution Transmission Electron Microscopy (HRTEM)

Bright-field transmission electronic images were taken using JEOL, JSM-3010 HRTEM, Japan, operated at 300 keV. Samples for TEM measurements were prepared by dropping the dispersion of the nanoparticles on a copper grid supported Formvar Films.

### *In vitro *Release Studies of Flurbiprofen SLN and NLC

*In vitro *release studies were performed using modified Franz diffusion cell. Dialysis membrane (purchased from Hi-Media, Mumbai, India) having pore size 2.4 nm, molecular weight cut-off between 12,000–14,000 was used. Membrane was soaked in double distilled water for 12 h before mounting in a Franz diffusion cell. Phosphate buffer pH 5.6 containing 0.5% w/v of polysorbate 80 was used as release media. SLN/NLC dispersion (1 ml) was placed in the donor compartment and the receptor compartment was filled with 0.5% polysorbate 80 in phosphate buffer, pH 5.6 (12 ml). During the experiments, the solution in receptor side was maintained at 37°C ± 0.5°C and stirred at 800 rpm with Teflon-coated magnetic stirring bars. At fixed time intervals, 100 μl of the sample was withdrawn from receiver compartment through side tube and analyzed by HPLC.

Data obtained from *in vitro *release studies were fitted to various kinetic equations to find out the mechanism of flurbiprofen release from FLUSLN and FLUNLC [6,7]. The kinetic models used were zero order equation, first order equation, Higuchi release and Korsemeyer-Peppas.

### Skin membrane preparation

The abdominal hair of Wister male rats, weighing 160 ± 25 g, was shaved using razors 24 h before treatment. After anesthetizing the rat with ether, the abdominal skin was surgically removed from the animal, and adhering subcutaneous fat was carefully cleaned. To remove extraneous debris and leachable enzymes, the dermal side of the skin was in contact with a saline solution for 1 h before starting the diffusion experiment. All surgical and experimental procedures were reviewed and approved by the animal and ethics review committee of Faculty of Pharmaceutical Sciences, Kakatiya University, Warangal, Andhra Pradesh, India.

### Ex Vivo Permeation studies

A system employing improved Franz diffusion cells with a diffusional area of 3.56 cm^2 ^was used for permeation studies. The excised rat skin was set in place with the stratum corneum facing the donor compartment and the dermis facing the receptor compartment. Two ml of SLN/NLC dispersion/0.5 g of the gel of flurbiprofen were applied to the skin surface in the donor compartment and the receptor compartment of the cell was filled with 12 ml of saline phosphate buffer (pH 7.4). During the experiments, the solution in receptor side was maintained at 37 ± 0.5°C and stirred at 800 rpm with Teflon-coated magnetic stirring bars. After application of the test formulation on the donor side, 100 μl aliquots were collected from the receptor side at designated time intervals (1, 2, 4, 8, 12, 18 and 24 h). Thereafter, an equivalent volume of receptor fluid was supplied to the receiver compartment immediately after each sample collection. At the end of 24 h, the amount of drug remaining on the skin and the drug concentration in the skin was determined by extraction into a suitable solvent followed by HPLC analysis.

### Rheological Measurements

The rheological measurements were performed on a rheometer Brookfield Programmable Rheometer LVDV-III + CP 230 equipped with a cone and plate test geometry (plate diameter 20 mm, cone angle 4°). All measurements were carried out at a temperature of 20 ± 0.1°C. The rheological properties of the developed hydrogels containing SLN and NLC were studied by continuous shear investigations, which were performed in order to evaluate the shear rate [1/s] as a function of shear stress [Pa]. This study started applying 0 Pa up to a maximum shear stress of 50 Pa and the resulting shear rate was measured.

### *In vivo *Studies

The animals used for *in vivo *experiments were adult male Wistar albino rats (230–250 g) procured from the central animal house of University college of Pharmaceutical Sciences, Kakatiya University, Warangal, Andhra Pradesh, India. The animals were kept under standard laboratory conditions, at 25 ± 1-C and 55 ± 5% relative humidity with a 12 h light/dark cycle. The animals were housed in polypropylene cages, with free access to a standard laboratory diet (Lipton Feed, Mumbai, India) and water. The study was approved by the Institutional Animal and Ethics Committee (Faculty of Pharmaceutical Sciences, Kakatiya University, Warangal, Andhra Pradesh, India). Guidelines of the institutional animal ethics committee were followed for *in vivo *experiments.

### Pharmacokinetic Studies of SLN and NLC enriched Hydrogels on Animals

The protocol for the studies was approved by institutional animal ethical committee. Wistar albino rats were used as the animal models for the bioavailability studies. The animals were selected after superficial examination of the skin surface for abnormalities. Only rats weighing between 230 and 250 g were selected for the study. About 9 cm^2 ^of skin was shaved on the dorsal side. Before application of the gels, rats were kept under observation for 24 h for any untoward effects of shaving; they were fasted over this period. The rats were divided into 3 groups (n = 6). Group I was administered flurbiprofen orally 10 mg/kg, 0.5 ml suspension in 0.5% sodium carboxymethyl cellulose solution in water, group II received SLN enriched hydrogel (A1), and group III received NLC enriched hydrogel (B1). The blood samples (250 μl) were collected in eppendorf tubes containing EDTA drawn from the tail vein of the rat at different time intervals (1, 2, 3, 4, 8, 12, and 24 h). Plasma samples were separated by centrifugation at 3000 rpm for 5 min (Micro Centrifuge, Kunchun, Korea) and stored in vials at -20°C until they were analyzed by HPLC. The results were compared with the orally administered flurbiprofen.

### Pharmacokinetic Analysis

Plasma concentration versus time data for flurbiprofen in individual rats was analyzed by using Kinetica (version 1.1). The statistical significance of the differences between the formulations was analyzed by Student t test using Graph Pad InStat 3 software. A difference below the probability level of 0.05 was considered statistically significant. Pharmacokinetic parameters C_max_, T_max_, t_1/2_, AUC_0–24 _and AUC_0-∞ _were estimated. The pharmacokinetic parameters estimated for each rat individually and average of six values was calculated.

### Pharmacodynamic Studies

Carrageenan induced paw edema method was used to study the *in vivo *performance of the prepared drug delivery system, and the study was approved by University Animal Ethical Committee. Anti-inflammatory activity was determined by measuring change in the volume of inflamed paw, produced by injection of carageenan (0.1 ml of 1% w/v) using plethysmometer (INCO, India). Male albino rats (Wistar strain) selected for the study were weighed and marks were made on the right hind paw just behind tibia-tarsal junction on each animal. Thus, every time the paw was dipped in the plethysmograph (mercury displacement method) up to the fixed mark to ensure constant paw volume. Wistar rats were divided into four groups including one controlled group with each group comprising of 6 animals. The paw volume was noted at 0, 1, 2, 4, 6, 8, 12 and 24 h.

The formulations A1 and B1 were applied transdermally to albino rats of respective groups, excluding the animals of controlled group. The controlled group animals were injected with saline (0.9% NaCl) containing no drug. After 30 min of transdermal application of A1 and B1 formulations, 0.1 ml of 1% w/v carrageenan (in 0.9% normal saline) was injected in the sub planter region of the right hind paw of rats. The initial reading just after injection and subsequent paw volumes was measured up to 24 h. The percent inhibition of edema induced by carrageenan was calculated for each group using the following equation:

$$\%\;inhibition\;of\;edema\;=\frac{V_{control\;\;}-\;V_{treated}}{V_{control}}\times 100$$

where, V_control _= mean oedema volume of rats in controlled group and V_treated _= edema volume of each rat in test group.

## Results and Discussion

### Characterization of the investigated formulation

For the production of aqueous FLUSLN and FLUNLC dispersions containing 20% of lipid matrix, the formulations given in table 1 were chosen.

**Table 1:** Composition of the investigated FLUSLN and FLUNLC formulations (% w/w)

**Formulations**	**Flurbiprofen**	**Dynasan 114**	**Epikuron 200**	**Captex 355**	**Polysorbate 80**
A (FLUSLN)	1.0	10	2	-	2.5
B (FLUNLC)	1.0	7	2	3	2.5

For the present investigation, four different hydrogels were prepared using optimal stabilizer combination of water, gel forming polymer and glycerol as hydrating agent. The aqueous FLUSLN and FLUNLC dispersions were admixed to the freshly prepared hydrogels. The final composition of the investigated FLUSLN and FLUNLC containing hydrogels were shown in table 2.

**Table 2:** Final composition of the investigated A (FLUSLN) and B (FLUNLC) enriched hydrogel formulations [% w/w]

**Formulations**	**Flurbiprofen**	**Dynasan 114**	**Captex 355**	**Epikuron 200**	**Polysorbate 80**	**Gel forming agent**	**Glycerol**
A1	1	10	-	2	2.5	1% Carbopol 934	5
A2	1	10	-	2	2.5	1% Xanthan gum	5
A3	1	10	-	2	2.5	2% HPC	5
A4	1	10	-	2	2.5	1% Chitosan	5
B1	1	7	3	2	2.5	1% Carbopol 934	5
B2	1	7	3	2	2.5	1% Xanthan gum	5
B3	1	7	3	2	2.5	2% HPC	5
B4	1	7	3	2	2.5	1% Chitosan	5

### Physicochemical properties

The FLUSLN and FLUNLC dispersions were light yellowish in colour, odourless and fluid in nature. It was stable and did not show sedimentation even after centrifugation. Gels loaded with FLUSLN and FLUNLC dispersions were light yellow in colour, odourless with smooth appearance.

### Particle size analysis

**Figure 1:**
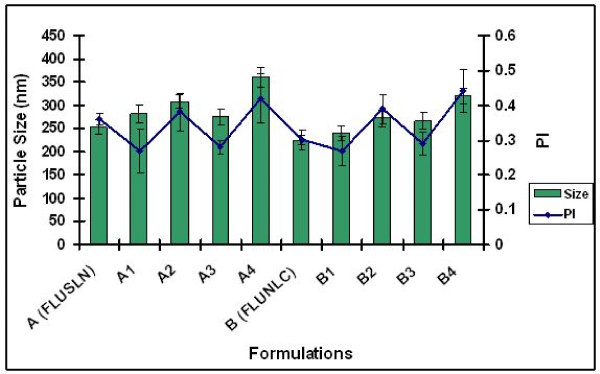
**Particle size analysis of FLUSLN and FLUNLC formulations after day 1 of storage at room temperature Mean, S.D (n = 3)**.

**Figure 2:**
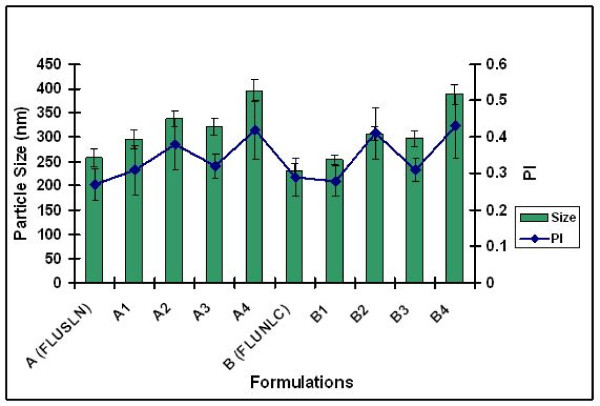
**Particle size analysis of FLUSLN and FLUNLC formulations after day 90 of storage at room temperature Mean, S.D (n = 3)**.

The particle sizes of lipid nanoparticles before and after incorporation into four different hydrogels were shown in figures 1 and 2. The P.I for Xanthan gum was approximately 0.3–0.4 and for chitosan hydrogel the P.I was approximately 0.4. After analysis the formulations were stored at room temperature for 90 days. For all the tested formulations more than 50% of the particles were below 250 nm after 90 days of storage at room temperature. The incorporation into hydrogels did not result in particle aggregation.

### Zeta potential (ζ)

FLUSLN and FLUNLC were negatively charged when incorporated into carbopol 934, xanthan gum and HPC hydrogels. The opposite was observed when incorporated into chitosan hydrogels. These results are due to the cationic character of the bioadhesive polymer. For carbopol 934 hydrogels, the carboxylic groups have to be neutralized with sodium hydroxide in order to exhibit gel forming properties. This neutralizing agent could enhance aggregation because of the action of sodium ions as electrolyte, which can reduce the ζ values of the particles [8]. As a consequence of this lower ζ value aggregation may occur. This phenomenon is well known for lipid nano emulsions as well as for SLN when incorporated into polyacrylate hydrogels [9]. The ζ values of the aqueous FLUSLN and FLUNLC dispersions before and after their incorporation into hydrogels measured on day 1 and after 90 days of storage at room temperature are given in table 3. During storage time, the ζ value of the surface lipid nanoparticles remains practically unchanged (e.g. B2, A1, and B1) or slightly decreased (e.g. A2, A3). In comparison to FLUSLN formulations with same liquid content, FLUNLC formulations show lower ζ values. FLUSLN and FLUNLC aqueous dispersions can be incorporated into hydrogels consisting of more or less uncharged polymers without significant changes in the particle size characteristics and ζ values [4]. In case of gel forming polymers with very polar groups like chitosan, possible interactions between negative surface charge of the lipid nanoparticles and polar groups of this polymer must be taken into account.

**Table 3:** Zeta potential and SD values of FLUSLN and FLUNLC before and after their incorporation into hydrogels measured on day 1 and day 90 of storage at room temperature

**Formation**	**Day 1**		**Day 90**	
	**ζ**	**S.D**	**ζ**	**S.D**
A (FLUSLN)	-28.3	3.65	-25.7	3.67
A1	-21.2	2.58	-20.2	6.54
A2	-34.2	6.53	-24.7	6.76
A3	-19.7	3.72	-15.4	4.74
A4	42.6	8.63	36.8	8.02
B(FLUNLC)	-21.7	3.98	-20.5	4.99
B1	-18.9	4.11	-18.6	6.34
B2	-20.5	5.65	-19.4	7.21
B3	-16.6	4.23	-14.6	3.89
B4	40.9	7.48	32.3	8.21

A (FLUSLN) and B (FLUNLC) before incorporation into hydrogels

### Assay and Entrapment Efficiency

A high amount of flurbiprofen could be incorporated in the SLN and NLC dispersions. As high as 30% of the drug with respect to the lipid could be incorporated, such high incorporation is possible because of the lipophilic character of flurbiprofen. Assay results showed that concentration of flurbiprofen in the total system ranged from 0.92 to 0.99 mg/ml.

The percentage of incorporated drug in the lipid matrix (entrapment efficiency) was evaluated over a period of 90 days. Incorporation of flurbiprofen led to high entrapment efficiency, probably because of their lipophilic character. FLUNLC is responsible for higher entrapment efficiency in comparison to FLUSLN formulation. This result is due to the binary mixture of liquid and solid lipids, resulting in only a very weak crystallization [10,11]. For all the tested formulations the entrapment efficiency was higher than 90%.

### Differential Scanning Calorimetry (DSC)

**Figure 3:**
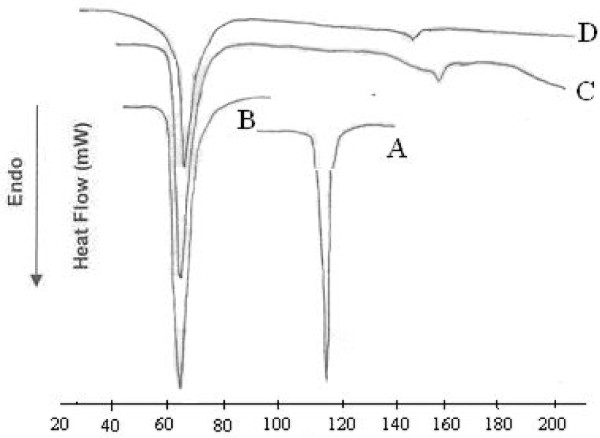
**DSC thermograms of flurbiprofen (A), trimyristin (B), physical mixture of flurbiprofen and trimyristin (C) and lyophilized flurbiprofen SLN (D)**.

**Figure 4:**
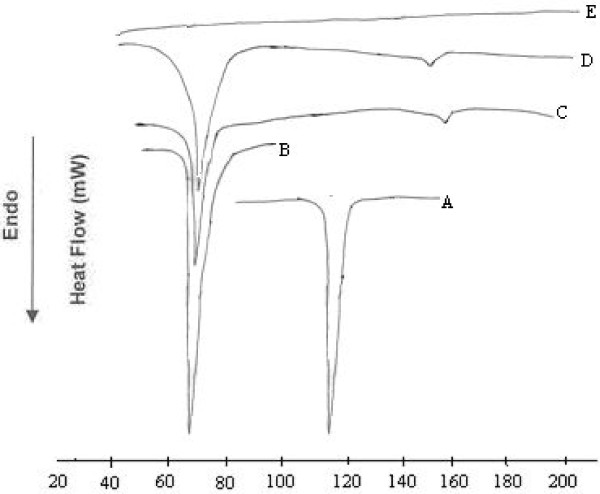
**DSC thermograms of flurbiprofen (A), trimyristin (B), physical mixture of flurbiprofen and trimyristin (C) lyophilized flurbiprofen NLC (D) and captex 355 (E)**.

Figures 3 and 4 shows DSC curves of flurbiprofen (FLU), trimyristin (TM), physical mixture (PM), lyophilized FLUSLN and lyophilized FLUNLC respectively. The thermograms of the lyophilized FLUSLN and FLUNLC did not show the melting peak for the flurbiprofen around 117°C. This shows that flurbiprofen was not in crystalline state but it is in amorphous state. Endothermic peak of glucose used as cryoprotectant was observed at 148.5°C in FLUSLN and FLUNLC curve. Similar results were reported by Cavalli et al. 1997, stating that rapid quenching of the microemulsion does not allow the drug to crystallize [12]. DSC analysis of camptothecin SLN prepared by high pressure homogenization showed that camptothecin was in amorphous state [13]. In our method, lipids and flurbiprofen were dissolved in a mixture of solvents and subsequently, solvents were evaporated. This allowed homogeneous dispersion of drug in the lipid. Furthermore, method of preparation (homogenization followed by ultrasonication) and the presence of surfactants do not allow the drug to crystallize. Thermodynamic stability of lipid nanoparticles depends upon their existing lipid modification. Polymorphic transitions after crystallization of triglyceride nanoparticles are slower for longer chain triglycerides than for shorter chain triglycerides, whereas these transitions are faster for small size of crystallites. The type of surfactant and storage time affects the crystallinity of SLN/NLC and, consequently, degradation velocity [14]. Melting points of lyophilized SLN and NLC were found to be 56.25°C and 57.25°C respectively.

### High Resolution Transmission Electron Microscopy (HRTEM)

**Figure 5:**
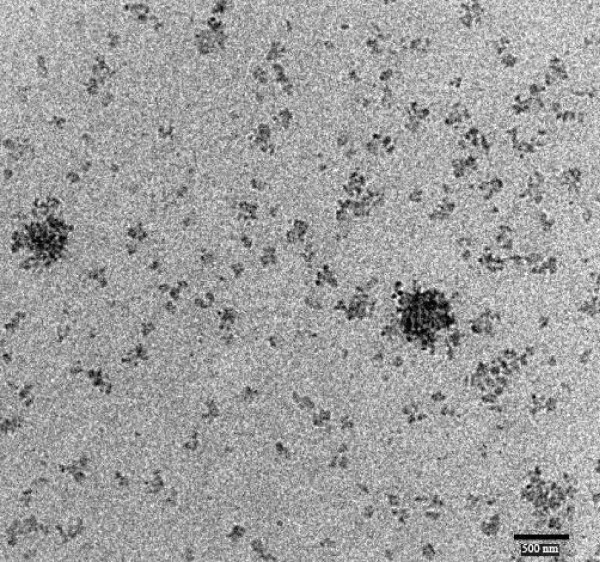
**TEM image of flurbiprofen solid lipid nanoparticles (FLUSLN)**.

**Figure 6:**
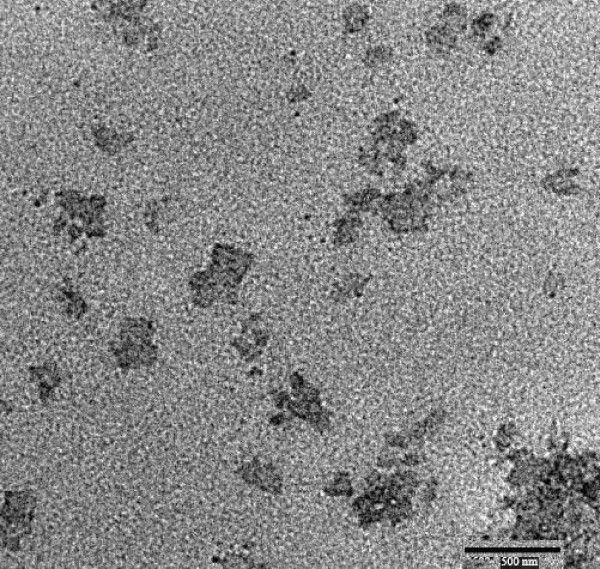
**TEM image of flurbiprofen nanostructured lipid carrier (FLUNLC)**.

TEM analysis has been performed to evaluate the potential differences in particle shape and morphology of SLN and NLC formulations. By this analysis the absence of aggregation phenomena of trimyristin based SLN and NLC has also been confirmed immediately after production, and also by monitoring the particle growth during storage time at room temperature. The TEM image of the FLUSLN and FLUNLC were shown in figure 5 and 6 respectively. Both the figures show that the particle diameters vary from about 150 to 300 nm. Both SLN and NLC were investigated, no significant difference in the diameter was obtained. TEM analysis suggested that immediately after production SLN and NLC formulations contained no particles higher than 1 μm.

### *In vitro *Release Studies

**Figure 7:**
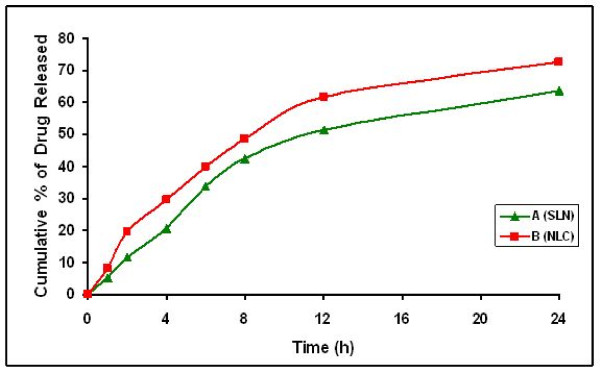
*In vitro *release profile of FLU in SLN and NLC dispersions, Mean S.D (n = 3)

The cumulative percentage release of flurbiprofen from A (FLUSLN) and B (FLUNLC) dispersions were investigated for a period of 24 h; each sample was analyzed in triplicate. Figure 7 shows the *in vitro *release profile of A (FLUSLN) and B (FLUNLC) dispersions. A (FLUSLN) could prolong or retard the drug release by the fact that the drug molecules are entrapped in the solid lipid matrix.

**Figure 8:**
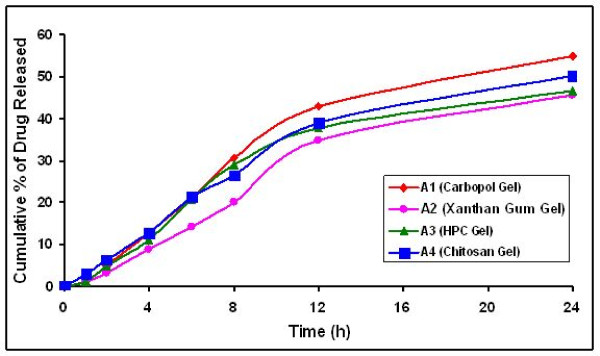
*In vitro *release of FLU from gels enriched with SLN dispersion, Mean S.D (n = 3)

**Figure 9:**
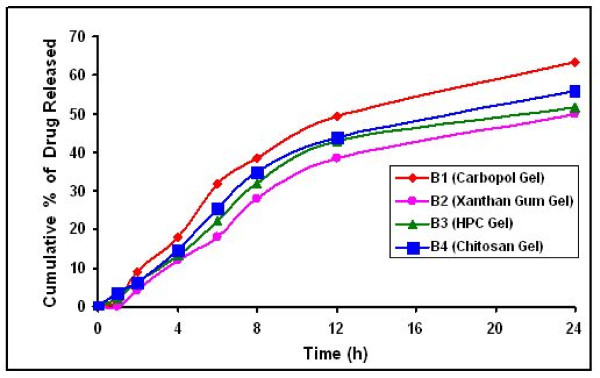
*In vitro *release of FLU from gels enriched with NLC dispersions, Mean S.D (n = 3)

FLUNLC, the liquid lipid enriched shell possessed soft and considerable higher solubility for lipophilic drugs character, in which the drug was easily loaded to higher amount and the drug could be easily released as well by the drug diffusion or the matrix erosion manners [15]. Furthermore, the incorporation of liquid lipid into solid lipid matrix caused the FLUNLC become more imperfect and allowed the loaded drug to easy release, thus increased the drug release rate when liquid lipid was included in NLC matrix. The results of sustained release and increased drug release rate were achieved compared to FLUSLN. Comparing the drug release from FLUSLN and FLUNLC dispersions and FLUSLN and FLUNLC in gels (figures 8 and 9), the release of FLU was slower from gel formulation. The percentage drug release at the end of 24 h in A1 (FLUSLN) and B1 (FLUNLC) gel formulations was found to 54.87% and 64.45% respectively whereas the percentage drug release at the end of 24 h in A (FLUSLN) and B (FLUNLC) dispersions was 63.66% and 72.65% respectively. Incorporation of SLN and NLC dispersion into gels decreased the drug release; this may be due to the release retarding effect of the polymeric matrix of the gelling agent.

A different release kinetic was observed for the SLN dispersions and SLN gel formulations. Fick's law of diffusion seems not to be applicable in each case. An initial rapid drug release was noted in the SLN/NLC dispersions, whereas a lag time was observed with SLN/NLC enriched gel formulations which could result from the time taken by the drug to diffuse across the gel. The direct exposure of SLN/NLC dispersion to diffusion media and quick release of drug may account for rapid initial release in SLN/NLC dispersions. Both SLN/NLC dispersions and SLN/NLC enriched gel formulations showed controlled drug release and also an increase in release rate was observed after 24 h.

The log percent cumulative drug released was plotted as a function of log time and the slope of the curves was determined as the values of diffusional release exponent (η). The values of diffusional release exponent (η) from the straight lines were noted to be 0.69–0.82 in SLN dispersions and SLN gel formulations, which showed that the release of drug from formulations followed a non-Fickian pattern [16]. From the percent cumulative drug released versus time plot, the slope values were determined as release rate constants. The percent cumulative drug released for B (FLUNLC) 72.65% and A (FLUSLN) 63.66% with release rate constants of 2.9515 and 2.691%/cm^2^/h, respectively. B1 (NLC) (63.45%) and A1 (SLN) gel (54.85%) have minimum drug release with release rate constants of 2.7527 and 2.4488%/cm^2^/h, respectively. A1 and B1 gel slowly releases the drug as compared with SLN and NLC dispersion, accounted for by the time the drug takes to diffuse through gel. The slower release of drug from SLN and NLC enriched carbopol gel maintained the drug concentration for longer period of time. Burst releases as well as sustained release both are of interest for dermal application. Burst release can be useful to improve the penetration of drug. Sustained release supplied the drug over a prolonged period of time.

### *In vitro *Skin permeation studies

**Figure 10:**
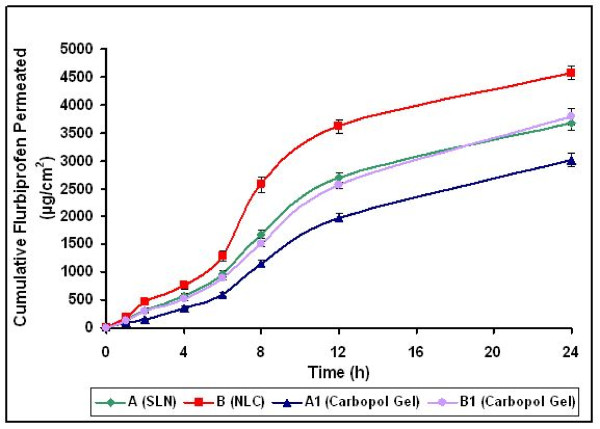
*Ex vivo *permeation of flurbiprofen from SLN and NLC enriched hydrogels through rat abdominal skin, Mean S.D (n = 3)

The *in vitro *skin permeation of flurbiprofen SLN and NLC was investigated through rat skin using Franz diffusion cell were shown in figure 10. B1 formulation hydrogel (area of 3.56 cm^2^) exhibited the greatest (3792.54 ± 149.98 μg/cm^2^) cumulative amount of drug permeation in 24 h than A1 formulation. The release kinetics was established by determining the diffusional release exponent from the plot of log of cumulative drug permeated versus log time. This plot yielded a straight line from which diffusional release exponent (n) was calculated and found to be between 0.89 – 0.96 for both SLN/NLC dispersions and SLN/NLC carbopol gel formulations, which showed that the release of drug from these formulations followed a non-Fickian pattern [16,17]. A lag time (15–30 min) was observed in every case but more in SLN/NLC gel formulations because in these formulations the drug has to cross two diffusion barriers, one the gel and the other is skin. The SLN/NLC dispersions and SLN/NLC enriched gel formulations possessed a sustained drug release over a period of 24 h period. But this sustained effect was more pronounced with SLN/NLC enriched gel formulations when compared to SLN/NLC dispersions.

From the percent cumulative drug permeated versus time plot, the slope values were determined as the skin permeation rate. The cumulative amount of drug permeated at the end of 24 h was found to be 3671.76 ± 128.82 and 4578.89 ± 116.16 μg/cm^2 ^with skin permeation rate constants of 7.8651 and 9.7519 percent/cm^2^/h for A (FLUSLN) and B (FLUNLC) dispersions respectively. The cumulative amount of drug permeated at the end of 24 h was found to be 3019.27 ± 126.19 and 3792.54 ± 149.98 μg/cm^2 ^with skin permeation rate constants of 5.9897 and 7.7954 percent/cm^2^/h for A1 and B1 enriched carbopol gels respectively. SLN and NLC enriched carbopol gels released drug slowly when compared with SLN and NLC dispersions, accounted for by the time drug takes to diffuse through the gel. The slower release of drug from SLN and NLC carbopol gels maintained the drug concentration for longer period of time.

The SLN/NLC dispersions and SLN/NLC gel formulations possessed a sustained drug release over a period of 24 h, but the sustained effect was more pronounced with SLN and NLC enriched gel formulations.

The results of drug permeation from all the formulations through the rat abdominal skin confirmed that flurbiprofen was released and permeated through the rat skin and hence could possibly permeate through the human skin.

### Rheological Measurements

Rheological measurements are useful for the characterization of the viscoelastic properties of aqueous SLN and NLC dispersions and SLN and NLC containing hydrogels. Therefore continuous shear investigations were performed in the tested hydrogel formulations in order to evaluate the shear rate as a function of shear stress. Conventional SLN and NLC aqueous dispersions contain about 10–20% (w/w) of lipid matrix and 80–90% (w/w) of water. As a result, liquid solid lipid dispersions possess a low viscosity (approximately 100 mPa s) and a yield value of practically zero. Therefore, the liquid solid lipid dispersions usually have to be incorporated in convenient topical dosage forms like hydrogels or creams to obtain a topical application form having the desired semisolid consistency [18,19].

**Figure 11:**
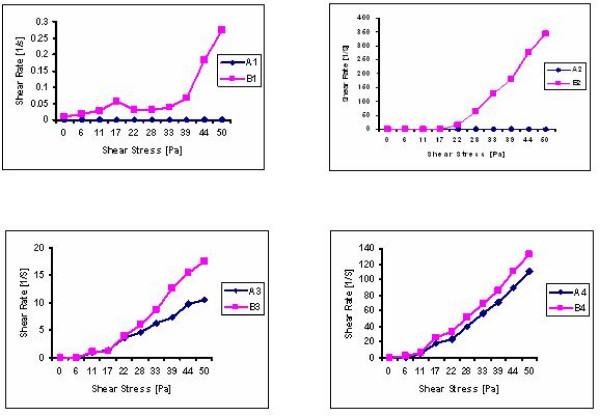
**Shear rates of SLN and NLC containing hydrogels as a function of shear stress, measured after 7 days of storage at room temperature**. (Composition of formulations: Table 2)

Rheological status is a very important physical parameter in the development of a potential new drug delivery system for topical use. The rheological behaviour of hydrogels loaded with SLN and NLC was evaluated after 1 week of storage at room temperature. The flow curves of the gels are shown in figure 11.

### Pharmacokinetic Studies of SLN and NLC enriched Hydrogels on Animals

**Figure 12:**
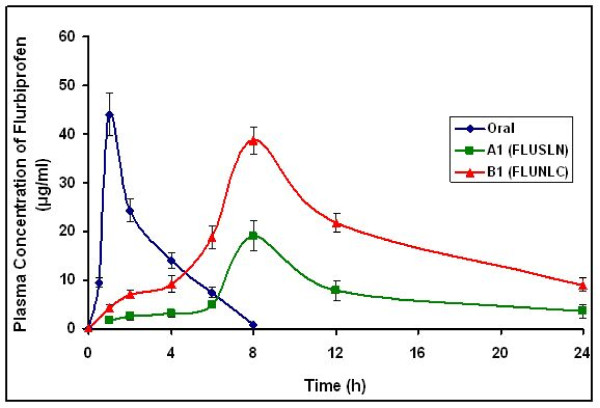
Plasma concentrations of flurbiprofen after oral and transdermal administration (n = 6)

Transdermal gels of 9 cm^2 ^area, containing 1 mg of the drug, were applied on the dorsal side of the rats. Figure 12 shows the plasma flurbiprofen concentration versus time profiles following the application of the hydrogel formulation. The time to reach maximum was 8 h for both A1 and B1 formulations. The C_max _of the B1 formulation, was 38.67 ± 2.77 μg/ml, which was significantly (P < 0.01) higher than the A1 formulation (C_max _= 21.79 ± 2.96 mg/ml). The C_max _and AUC of the B1 formulation were 1.8 and 2.5 times higher than the A1 formulation respectively (Table 4).

The AUC decreased in the following order A1 > B1 > oral. The pharmacokinetic parameters of flurbiprofen after the oral administration were significantly different from the parameters obtained after the transdermal application of gel formulations. After the oral administration of flurbiprofen, the C_max _of the drug reached within 1 h, and a sharp decline was observed as shown in figure 12.

The increase in the AUC_0–∞ _of flurbiprofen after application of transdermal gel formulations was significantly higher than orally administered flurbiprofen, which indicates the improved bioavailability of flurbiprofen through transdermal drug delivery route. The bioavailability of flurbiprofen with reference to orally administered flurbiprofen was found to increase by 4.4 times when transdermal gel formulations were applied. Greater flurbiprofen plasma levels were achieved immediately and were maintained till the last sample. In other previously reported study flurbiprofen levels of 43.9 μg/ml were achieved in rats at 1.8 h after the application of 500 μl of 1% flurbiprofen containing 5% of oleic acid and 5% urea in propylene glycol, on the dorsal area of 9 cm^2 ^[20]. In this study, higher plasma levels of flurbiprofen were achieved with the application of transdermal hydrogels.

### Pharmacodynamic Studies

The *in vivo *performance of selected A1 (FLUSLN) and B1 (FLUNLC) enriched hydrogel were carried out in carrageenan-induced rat paw edema model. Both the formulations A1 and B1 under study not only decreased the inflammation to the larger magnitude, but also sustained this magnitude. In A1 formulation the maximum inhibition was observed at 8^th ^h with higher value (78.2%), up to 8 h inhibition was maintained above 60%, and even after 24 h, 35% inhibition was observed.

**Figure 13:**
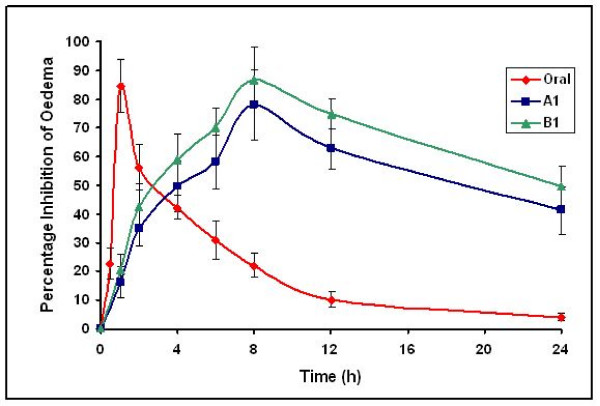
Anti-inflammatory activity of flurbiprofen SLN and NLC enriched hydrogels after transdermal application in comparison to oral administration in carrageenan induced rat paw oedema (n = 6)

In B1 formulation maximum inhibition was observed at 8^th ^h with higher value (86.6%), above 70% inhibition was observed up to 8 h and more than 45% inhibition was observed even after 24 h. However, in case oral administration, inhibition was displayed at 1 h with magnitude of 84.6% and just after 4 h it scored below 40% (Figure 13). The possible reason could be the drug concentration in the blood, which was maintained for longer duration in case of formulations A1 and B1 in comparison to the drug administered orally. In comparison to orally administered flurbiprofen, the two formulations A1 and B1 which were applied transdermally gave good results. The maximum inhibition for A1 and B1 were observed at 8 h and the inhibition was maintained up to 12 h and also even after 24 h inhibition was observed. The anti-inflammatory activity of both the formulations (A1 and B1) was maintained for longer period of time due to slow release of the drug. This was attributed to gel structure and the surface-active properties of the gel.

## Conclusion

Both FLUSLN and FLUNLC represent a highly effective, non-irritant carrier for transdermal/topical preparations, where improved drug penetration is desired. Improved skin penetration can be due to enhanced contact of the active agent and skin resulting from the large particle surface area and film formation. After 90 days of storage at different temperatures the mean diameters of SLN and NLC remain practically the same (< 1 μm), which emphasizes the physical stability of these lipid particles. FLUNLC showed higher entrapment efficiency due to their liquid parts. FLUNLC also showed a faster release profile in comparison to FLUSLN. Both the SLN and NLC dispersions and gels enriched with SLN and NLC possessed a sustained drug release over period of 24 h but the sustained effect was more pronounced with the SLN and NLC gel formulations. The rheological investigations provide information about application of semisolid formulations and their performance on skin; these studies were conducted both on SLN and NLC enriched hydrogels. These systems are complex and their performance is dependent on structure of the gel forming polymer used for hydrogel preparation. Moreover, by increasing the solid lipid content of the dispersed phase an increase in elastic component was observed.

The pharmacokinetic parameters obtained with transdermal gels were significantly different from those obtained with oral flurbiprofen administration. The results indicate that the elimination half-life of flurbiprofen was prolonged from oral administration to the gel formulations in rats. This indicates that the drug remains in the body for a longer period and has a sustained action. The significantly high AUC values observed with transdermal gels also indicate increased bioavailability of the drug from gels compared with oral administration. Since the administration of flurbiprofen through gels resulted in sustained and continued drug release for 24 h, the gels were able to control the inflammation throughout the period. The anti-inflammatory activity of both the formulations (A1 and B1) was maintained for longer period of time due to slow release of the drug.

Clearly, the prepared transdermal gel formulations (A1 and B1) are capable of surmounting the shortcomings of oral administration of flurbiprofen, such as low bioavailability, short half-life, and high first pass metabolism.

## Competing interests

The authors declare that they have no competing interests.

## Authors' contributions

KB carried out the formulation, in vitro characterization and drafted the manuscript. JA carried out the in vivo studies. VR coordinated for the study. VV designed the protocol of the study. YMR participated in design and coordination of the study. All authors read and approved the final manuscript.
